# Management of Severe Riga-Fede Disease in a Child With MIRAGE Syndrome

**DOI:** 10.7759/cureus.97970

**Published:** 2025-11-27

**Authors:** Kolapo Dairo, Neil Mehta, Jonathan Grischkan, Ashok Kumar, Charlotte Sjulin

**Affiliations:** 1 Department of Otolaryngology, The Ohio State University College of Medicine, Columbus, USA; 2 Department of Otolaryngology, The Ohio State University Wexner Medical Center, Columbus, USA; 3 Department of Pediatric Otolaryngology, Nationwide Children’s Hospital, Columbus, USA; 4 Department of Pediatric Dentistry, Nationwide Children’s Hospital, Columbus, USA; 5 Department of Pediatric Dentistry, Pediatric and Adolescent Dentistry, Marysville, USA

**Keywords:** immunodeficiency, mirage syndrome, oral surgery, riga-fede disease, traumatic mucosal ulceration

## Abstract

Riga-Fede disease (RFD) is a rare condition in infants and young children caused by repetitive trauma to the oral mucosa, often from newly erupted teeth. We present the case of a 13-month-old male child with a complex genetic disorder who developed persistent ulceration on the ventral tongue due to contact with mandibular incisors.

Initial management included conservative smoothing of the teeth, which led to only partial improvement. Over time, the mucosal ulceration worsened, resulting in mucosal separation and soft tissue loss. A multidisciplinary surgical approach was pursued. Pediatric otolaryngology performed excision of the compromised mucosa, re-approximation of the muscular defect with absorbable sutures, and a lingual frenuloplasty to address ankyloglossia. Pediatric dentistry completed the extraction of the four mandibular incisors to eliminate the source of trauma.

This case illustrates the importance of early recognition and definitive treatment of RFD to prevent progression to more complex soft tissue defects. In medically complex patients, delays in intervention may lead to increased morbidity and impaired healing. Successful management requires close collaboration between specialties and careful surgical planning to preserve function and reduce recurrence risk.

## Introduction

Riga-Fede disease (RFD) is a benign and uncommon mucosal disorder of the oral cavity characterized by ulceration of the ventral tongue. This condition was first described by Riga in 1881 in Italy and then later characterized by Fede in 1890. This condition is often caused by injuries to the mucosa of the tongue due to repetitive and traumatic movements of the tongue over the mandibular anterior incisors [1]. This frequently results in increased irritability of patients related to pain and can lead to difficulties with oral feeding, dehydration, and growth restriction.

MIRAGE syndrome is an extremely rare genetic disorder characterized by Myelodysplasia, Infection, Restriction of growth, Adrenal hypoplasia, Genital phenotypes, and Enteropathy. As of 2020, only 44 individuals with this condition had been reported in the literature. Patients with MIRAGE syndrome can have a variety of symptoms related to the defining features, including cytopenias, hypospadias, hypoplastic ovaries, esophageal dysfunction, and developmental delay. Those afflicted with the disorder have a short life expectancy, with a median age of death at three years of age, typically secondary to infection [2].

We report a case of RFD in a child with MIRAGE syndrome. To our knowledge, this is the first reported case in the literature of a patient with both disorders.

## Case presentation

**Figure 1:**
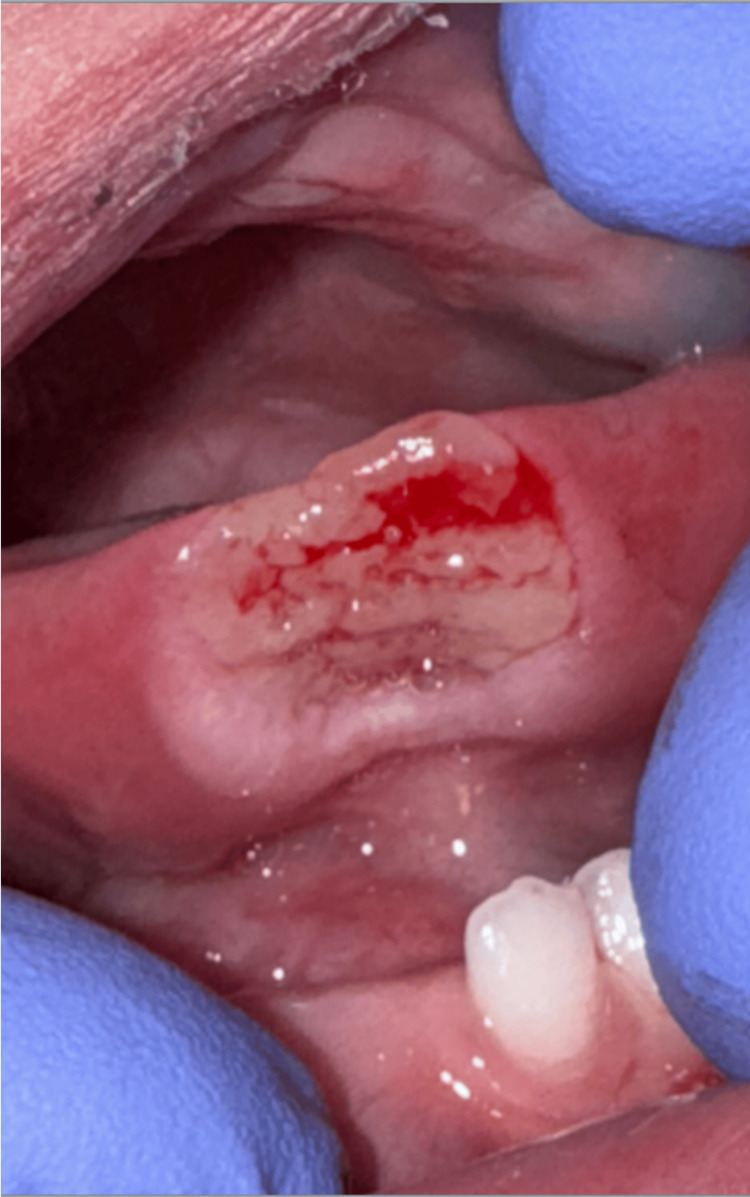
Initial dental consult exam revealed Riga-Fede disease A 16 mm X 8 mm ulceration on the ventral surface of the tongue and the two erupted mandibular primary central incisors were noted.

A 13-month-old male child was seen for oral ulcers, and a consultation was made to dentistry. The patient’s history was significant for premature birth, pancytopenia, and massive intracranial hemorrhage within days of birth. A comprehensive workup ultimately revealed a diagnosis of MIRAGE. The intraoral examination revealed two incisors in the anterior mandible and an ulceration on the ventral surface of the tongue (Figure 1). Further examination of the oral ulceration revealed a rectangular lesion estimated to be 16 mm by 8 mm in contact with the mandibular central incisors. No other lesions were appreciated on the intraoral mucosa. Based on the history and clinical features, a diagnosis of RFD was made. 

**Figure 2:**
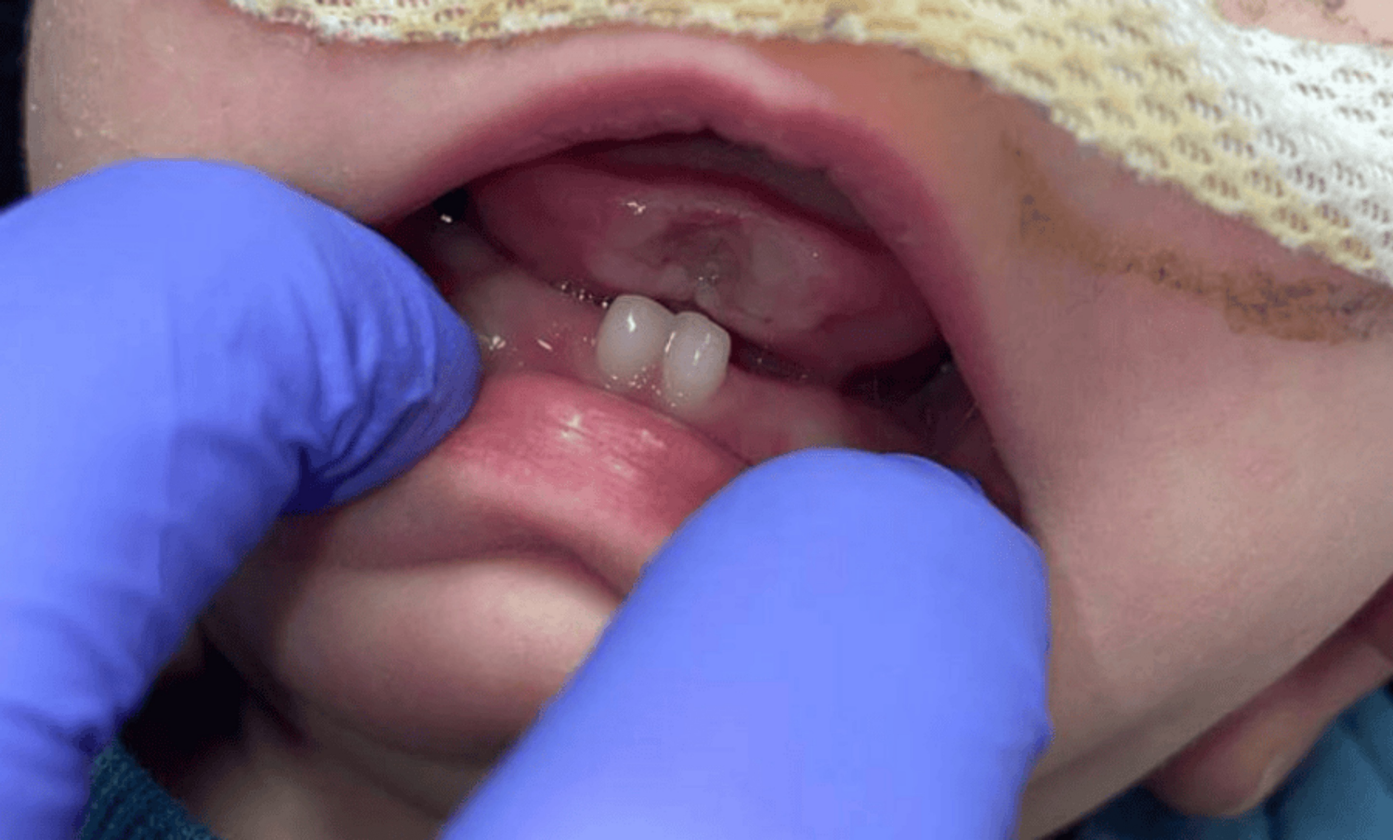
Two weeks after initial smoothing of the mandibular central incisors The tongue displayed only slight resolution of the ulceration.

**Figure 3:**
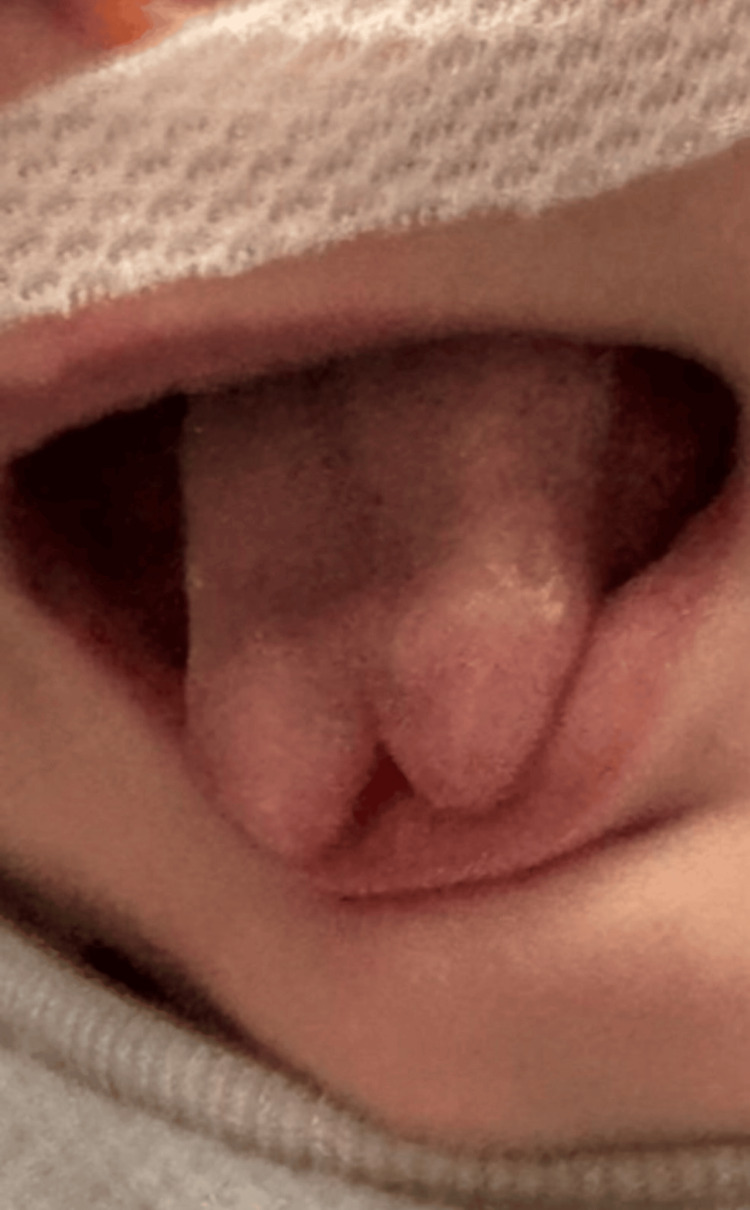
Significant tissue dysmorphia of the patient's tongue eight weeks after initial smoothing of the mandibular primary central incisors

Extractions of the mandibular primary central incisors, #O (lower left primary central incisor) and #P (lower right primary central incisor), were recommended as the treatment of choice given the extent of mucosal erosion; however, this was initially declined by the family. Rather, they preferred a conservative, non-surgical treatment approach. A smoothing procedure was performed using a sanding strip as well as a slow-speed handpiece with polishing discs. The procedure was completed using non-pharmacological behavior management techniques. After two weeks, the parents presented for a follow-up office visit and reported some relief in symptoms. At that time, the tongue revealed only slight resolution in the extent of ulceration (Figure 2), and further smoothing of the mandibular central incisors was indicated. The same non-pharmacological behavior management techniques were implemented, and the smoothing procedure was again performed using a slow-speed handpiece and polishing discs. Eight weeks following the initial smoothing procedure, the parents reported dissatisfaction with the aesthetics of the infant’s tongue. They had begun to notice a splaying of the anterior tip of the tongue (Figure 3). At this time, pediatric otolaryngology was consulted, and possible repair options were discussed with the family. Multidisciplinary discussions concluded that the teeth would need to be extracted for the tongue re-approximation to be successful long-term. With consent from the family, pediatric otolaryngology and pediatric dentistry performed a combined effort surgery.

**Figure 4:**
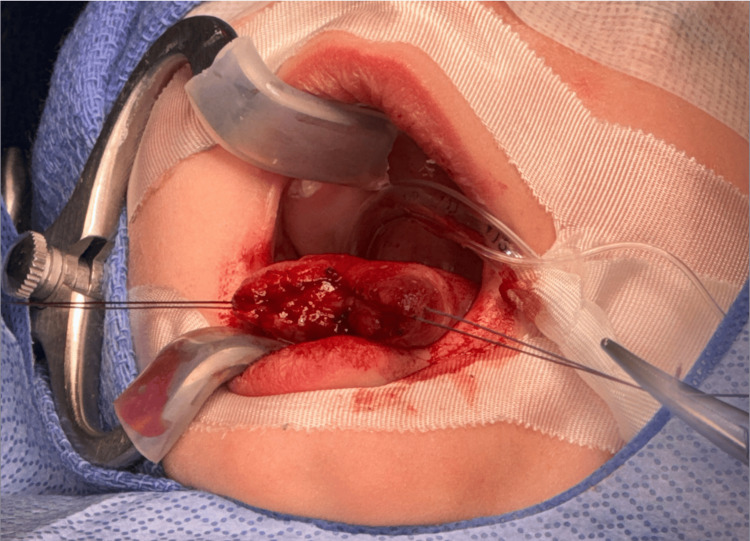
De-epithelialization of the defect, exposing the superficial mucosa of the tongue

**Figure 5:**
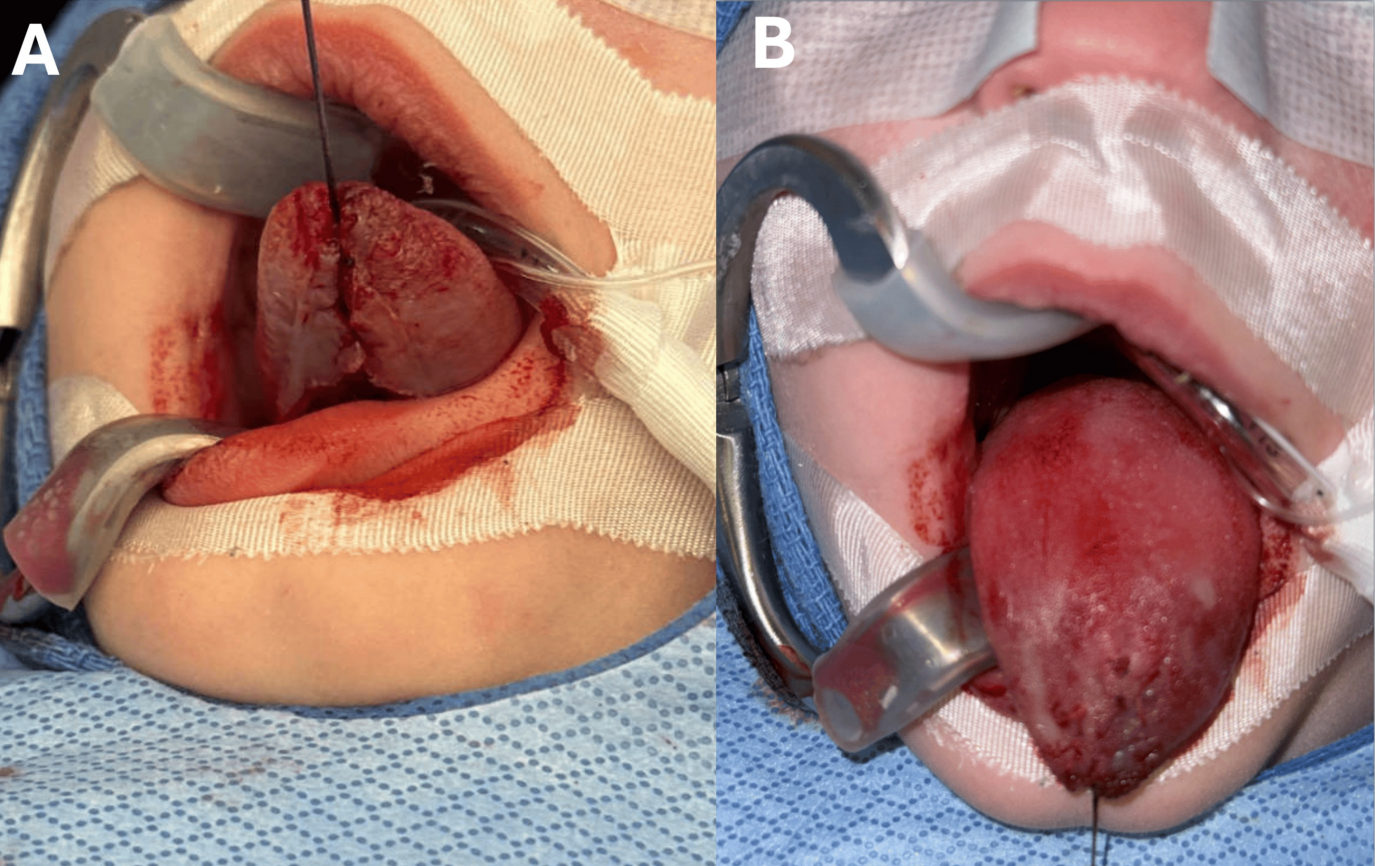
Inferior (A) and superior (B) view of the repaired defect

**Figure 6:**
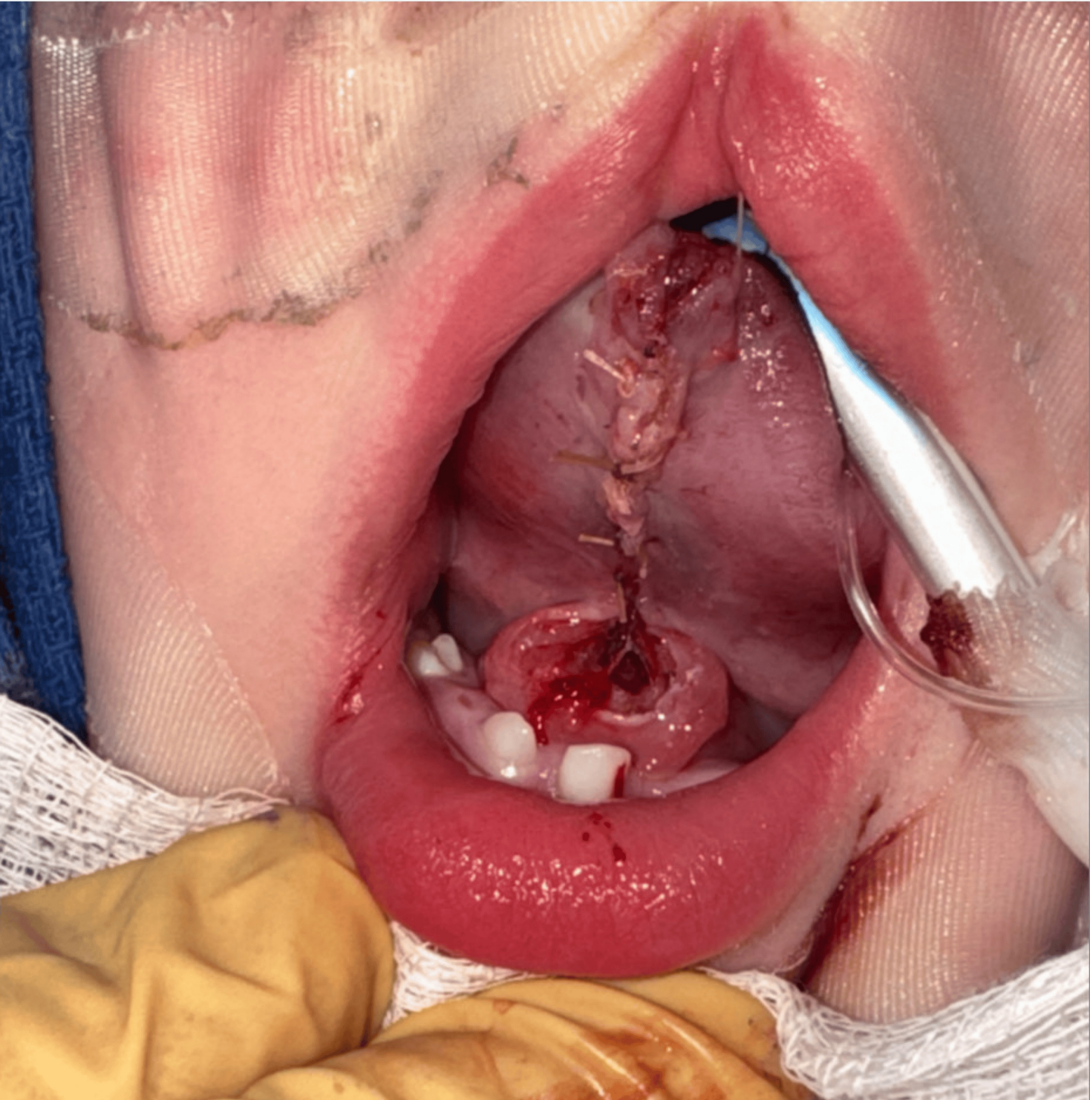
Release of the lingual frenulum provided improved tongue mobility

First, the pediatric otolaryngology team proceeded with the repair of the tongue defect. The procedure was performed in the operating room under general anesthesia. Mucosal incisions were performed sharply with a 15-blade scalpel to freshen the edges of the anterior tongue defect along the dorsal and ventral surfaces (Figure 4). The tongue defect was then repaired in a multilayered fashion. The mucosa was undermined on the dorsal and ventral surfaces to elevate from the genioglossus muscle, allowing the free edges of intrinsic tongue musculature to be re-approximated. The mucosal edges were then re-approximated using absorbable Vicryl sutures and a horizontal mattress technique (Figure 5). In addition, the patient was noted to have significant ankyloglossia. While this was not the primary cause, the abnormal anatomy may have been a contributing factor to the development of the trauma to the tongue, so a lingual frenuloplasty was performed to release the tongue and allow for improved mobility (Figure 6).

**Figure 7:**
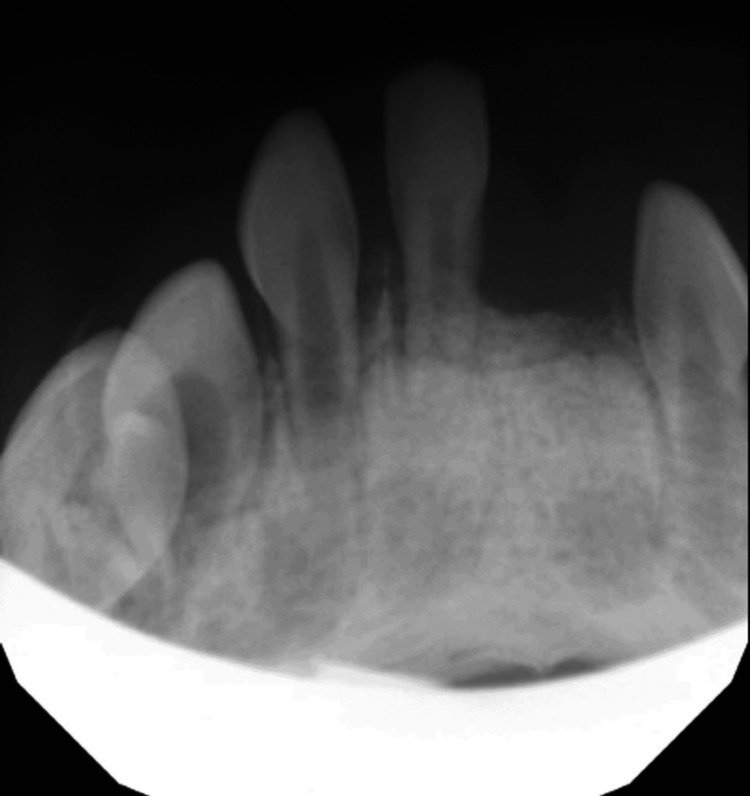
A mandibular occlusal radiograph was taken on the day of surgery

**Figure 8:**
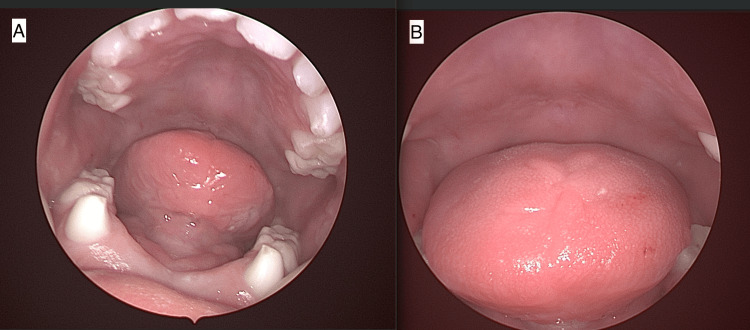
A follow-up visit at 17 months showed well-healed ventral (A) and dorsal surfaces of the tongue (B) and healed dental extraction sites on the mandibular alveolus

Pediatric dentistry then completed its portion of the procedure. Of note, #O (lower left primary central incisor) displayed excessive horizontal and vertical mobility (Class 3), so the tooth was extracted during the first portion of surgery to prevent interference with otolaryngology’s procedure. A single mandibular occlusal radiograph was obtained (Figure 7). A flap was raised to uncover and access #N (lower left primary lateral incisor) for extraction. #P (lower right primary central incisor) was extracted. A flap was raised to gain better access to #Q (lower right primary lateral incisor) for extraction. Two simple interrupted 5-0 resorbable chromic gut sutures were placed over the extraction sockets of #N and #Q for improved tissue approximation. The patient recovered from surgery without any complications. The patient continues to do well 17 months after surgery (Figure 8).

## Discussion

This case highlights RFD's unique presentation and emphasizes the challenging approach to management in a patient with a concurrent genetic syndrome. For this patient, the presence of two midline mandibular primary incisors posed a persistent source of irritation to the ventral tongue, ultimately resulting in ulceration and splaying of the anterior tip, which led to a significant forked-tongue deformity. 

The diagnosis of RFD is often clear based on history and clinical features, and an extensive workup is typically not necessary. Still, in patients with an abnormal mass or lesion, suspicion should always be high for more serious pathology. Van der Meij et al. described a case of RFD in a six-month-old infant who required biopsy of the lesion because the ulcerated mass looked highly concerning for malignancy [3]. Although the lesion turned out to be benign, histopathology played a role in ruling out more sinister disease. In our case, a biopsy was not necessary, as there was a clear connection between the mandibular primary incisors and the subsequent ulceration.

Traditional treatment options for RFD range from conservative to surgical. A watch-and-wait approach may be considered in mild disease, and modification of feeding behaviors can provide additional benefit. Kenalog in Orabase, a topical medication with anti-inflammatory and local analgesic effects, has utility in symptom relief [4]. In those with refractory or more severe disease, smoothing of the incisal edges or extraction of the involved teeth can provide relief from the source of trauma. In this patient, initial attempts at tooth reshaping with sanding and polishing yielded only partial improvement. The delay in definitive management contributed to continued tissue damage and progression to a more complex tongue defect requiring surgical intervention. 

An important factor that delayed prompt definitive treatment for this patient was parental anxiety regarding a surgical approach. The parents expressed significant hesitancy when tooth extraction was initially recommended due to concerns for a negative cosmetic outcome. Anxiety for parents of children having surgery is greater in children of younger age and/or undergoing larger-sized surgeries, and it can be difficult for parents to cope with the altered appearance of their child [5]. It was not until there was already a significant cosmetic defect that the severity of the RFD was understood, necessitating surgical management. A mandibular occlusal radiograph should be taken to confirm the presence of succedaneous teeth, as done in this case. Parents of infants with RFD can then be reassured that the child will only be missing teeth in the lower anterior region until the permanent successors erupt, typically around six years of age. 

The surgical approach required precise planning to address the tongue defect while preserving function. Excision of the compromised mucosa and re-approximation of the tongue musculature successfully restored the integrity of the tongue. Removal of the mandibular primary incisors addressed the etiology of the oral trauma, reducing the long-term risk of recurrence. The concurrent lingual frenuloplasty and genioglossus release was a strategic addition, improving future tongue mobility and addressing a possible contributing factor. 

The presence of MIRAGE syndrome complicated our management strategy. Patients with this syndrome can demonstrate immunodeficiency and poor nutritional status, which may compromise adequate wound healing and predispose them to wound dehiscence and postoperative infection [2]. Because of the rarity of each of these disorders, there is no prior literature describing patients who have both. Thus, these considerations necessitated close coordination between pediatric otolaryngology and pediatric dentistry to ensure optimal perioperative timing, comprehensive treatment, and close follow-up. The involvement of multiple specialties highlights the value of collaborative multidisciplinary approaches in managing complex pediatric cases with rare genetic conditions.

## Conclusions

This case emphasizes the importance of early diagnosis and proactive management in preventing complications associated with RFD, particularly in patients with complex secondary medical conditions. The successful outcome was achieved through a combination of surgical management, multidisciplinary collaboration, and shared decision-making with the family. By addressing both the immediate pathology and potential contributing factors, this case underscores the need for comprehensive care strategies. Ultimately, timely intervention and coordinated care played a crucial role in ensuring a positive outcome for this patient. By combining clinical judgment and patient-centered care, this case demonstrates a successful outcome in a challenging clinical scenario.
